# Nurses’ Perception of, and Barriers to, Delivering Cardiopulmonary Rehabilitation for Heart Failure Patients: A National Survey in Saudi Arabia

**DOI:** 10.3390/ijerph192013586

**Published:** 2022-10-20

**Authors:** Abdulelah M. Aldhahir

**Affiliations:** Respiratory Therapy Department, Faculty of Applied Medical Sciences, Jazan University, Jazan 45142, Saudi Arabia; aldhahir.abdulelah@hotmail.com

**Keywords:** CR, heart failure, cardiopulmonary rehabilitation, nursing, Saudi Arabia

## Abstract

Background: Heart failure (HF) patients require a holistic management approach to improve their clinical outcomes. Cardiopulmonary rehabilitation (CR) is a core component of HF patients’ management and is supervised by a multidisciplinary team including nurses. Nursing attitudes regarding CR delivery for patients with HF and the possible barriers and factors that potentially affect referral have not been explored. Therefore, this study seeks to evaluate nurses’ attitudes towards the delivery of CR programs and the possible barriers and factors that potentially influence the decision for a referral. Methods: An online survey with eight multiple-choice items was disseminated to all nurses between February and July 2022 in Saudi Arabia. The characteristics of the respondents were described using descriptive statistics. Percentages and frequencies were used to report categorical variables. Results: Overall, 1056 nurses completed the online survey, of which 395 (37.40%) were male. Out of 1056 nurses, 414 (39.20%) strongly agreed that CR would improve patients’ physical fitness, and 392 (37.10%) strongly agreed that CR would reduce breathlessness in patients with HF. In total, 381 nurses strongly agreed (36.10%) that CR would improve HF patients’ palpitation and fatigue. Out of 1056 nurses, 396 (37.50%) strongly agreed that CR would improve HF patients’ ability to perform daily activities, and 326 (30.90%) strongly agreed that CPR would reduce the rate of hospital readmission. The at-home program was the preferred mode of delivering CR programs among 607 (57.50%) nurses. Apart from the exercise component, symptom management was perceived by 704 (66.70%) nurses as the fundamental component of CR programs. The most common patient-related factor that strongly affected the decisions regarding referral was “mobility affected by breathlessness” (57%). A lack of CR centers (46%) was the most common barrier. Conclusion: Nurses perceived CR as an effective management strategy for HF patients. Although a home-based program, with symptom management being an essential component, in addition to the exercise component, was perceived as the preferred mode of delivery, CR centers are lacking, which represented a significant barrier to CR referral from the nurses’ perspective.

## 1. Introduction

Heart failure (HF) is a severe clinical syndrome associated with symptoms and signs that result from the inability of the heart organ to deliver and pump sufficient blood, along with the necessary nutrients, in order to meet the human body’s requirements, leading to reduced organ perfusion and ultimately death unless it is treated appropriately [1]. Globally, HF is a cause of morbidity and mortality [2]. Exercise intolerance and functional impairment are among the common symptoms of patients with HF. These symptoms frequently worsen with exertion, leading to exacerbations and unnecessary emergency visits or hospitalization [1].

HF is incurable, unlike other diseases or bodily ailments, but pharmacologic and non-pharmacologic strategies may reduce the exacerbations and need for hospitalization [1]. Therefore, a holistic management approach, such as cardiopulmonary rehabilitation (CR), should be implemented to mitigate HF symptoms [3]. CR is an effective non-pharmacologic management strategy for individuals with HF. CR for patients with HF is a comprehensive, multidisciplinary program that includes the assessment of outcomes and exercise training, aiming to improve the patients’ functional capacity and quality of life [2]. The CR program should include a medical examination, patient education, nutritional support, mental health and psychosocial support, and a physical activity counselling program [3,4]. CR must be conducted by a multidisciplinary team that includes doctors, physiotherapists, psychologists, dietitians, social workers, and nurses [2,5].

In the past, the physical activity of patients with HF was believed to aggregate the disease. However, several studies have supported the idea that the participation of patients with HF in physical activity and exercise training is safe and well tolerated in stable HF patients [6,7,8,9]. Furthermore, studies have shown that CR can improve the functional ability, exercise duration, quality of life, peak oxygen consumption, and endothelial function, and reduce the need for hospitalization in individuals with HF [3,10,11,12,13,14]. Interestingly, CR services remain underutilized globally, including in Saudi Arabia [2,15,16]. To the best of our knowledge, there is only one CR program in Saudi Arabia specifically for patients with cardiovascular diseases, including HF [16]. Recently, we conducted a study to assess physiotherapists’ attitudes towards the delivery of CR to HF patients and to identify factors and barriers that might influence CR referral decisions from their perspective. Our findings showed that a supervised hospital-based program with stress management as an essential component was the preferred method of delivering CR, but CR centers were lacking [16]. To promote the implementation of the CR program in Saudi Arabia, it is essential to assess nurses’ perception of the delivery of CR programs for patients with HF, given that nurses are part of the CR team [17]. In addition, the barriers and factors that potentially affect decisions to refer HF patients to the CR program from the perspective of nurses should be considered. Therefore, the study aims to assess nurses’ perception of the delivery of the CR program to patients with HF and to identify possible barriers and facilitating factors that might potentially affect the referral decisions, specifically from the perspective of nurses in the Kingdom of Saudi Arabia.

## 2. Materials and Methods

### 2.1. Study Design

This cross-sectional study was carried out between 13 February and 22 July 2022. An online survey platform (Survey Monkey) was used to disseminate the survey and collet data.

### 2.2. Questionnaire Tool

We used a modified version of a questionnaire that was previously designed, created, and validated by Aldhahir et al. [17,18,19,20]. The questionnaire included eight closed-ended multiple-choice questions. Participants were informed of the study’s objective and the identity of the principal investigator before beginning the questionnaire. The survey began with a question asking the participants if they were interested and willing to participate in this study. The survey stated, “By answering yes in completing the survey question, you freely agree to engage in this study and offer your agreement to utilize your anonymous data for research purposes”. Three to five minutes were the expected duration required to complete the questionnaire. The first part collected data about demographic information. The second part asked participants about three domains, which were effectiveness, components, and delivery methods of CR. This section offered a 5-point Likert scale to enable responses to all the statements. The responses ranged from 1, “strongly disagree”, to 5, “strongly agree”. The last part asked participants about both patient and process-related factors that influence the referral decision regarding CR. Participants were asked to grade the factors. The options, in this part, were no influence, some influence, and a strong influence.

### 2.3. Study Population and Sampling Strategy

A convenience sampling strategy was used to recruit participants for this study. The target population were nurses who worked with HF cases in Saudi Arabia. To reach more professionals working in Saudi Arabia, the questionnaire was distributed through the nursing committee and social media platforms (Twitter, WhatsApp, Telegram). The criteria for inclusion in the study were clearly specified in the study invitation.

### 2.4. Sample Size

The design of the study was exploratory; therefore, a sample size calculation was not required.

### 2.5. Ethical Approval

Jazan University’s Institutional Review Board approved the study (reference number REC-43/03/041).

### 2.6. Statistical Analysis

The Statistical Package for Social Sciences (SPSS software, Version 25, IBM, Armonk, NY, USA) was used to analyze the data. The reported and displayed categorical variables used percentages and frequencies.

## 3. Results

Overall, 1056 nurses, including both male 395 (37.40%) and female 661 (62.20%) nurses, responded to the online survey between 13 February and 22 July 2022. The majority of respondents were from the central region (280, 26.51%) and the western region (320, 30.30%) (Table 1). A high percentage of nurses had one to two (26.20%) or three to four (24.20%) years of clinical experience in caring for patients with HF (Table 1). The most common responsibilities of the nurses in caring HF patients were urgent assessment (61.40%), followed by primary care (59.60%) and in-patient treatment (49.70%) (Table 1).

### 3.1. Opinion on Referring Patients with Heart Failure to Cardiopulmonary Rehabilitation

Out of 1056 nurses, 414 (39.20%) strongly agreed and 438 (41.50%) agreed that CR would improve patients’ physical fitness. Additionally, 392 (37.10%) strongly agreed and 427 (40.40%) agreed that CR would reduce breathlessness in patients with HF. The majority of nurses strongly agreed (381, 36.10%) or agreed (400, 37.90%) that CR would improve HF patients’ palpitation and fatigue. Out of 1056 nurses, 396 (37.50%) strongly agreed and 427 (40.40%) agreed that CR would improve HF patients’ ability to perform daily activities, and 326 (30.90%) strongly agreed and 368 (34.80%) agreed that CR would reduce hospital readmission (Table 2).

### 3.2. Mode of Delivery and Component of Cardiopulmonary Rehabilitation

Out of 1056 nurses, 607 (57.50%) believed that the preferred way to deliver a CR program is at home, followed by 571 (54.10%) who believed in hospital-supervised programs. (Table 3) In contrast, an online program with healthcare provider support was the least preferred way to deliver a CR program among 525 nurses (49.10%). 

Symptom management, followed by stress and weight management, were the essential components of a CR program from the nurses’ perspective, excluding the exercise component, according to 704 (66.70%), 698 (66.10%), and 691 (65.40%) nurses, respectively (Table 3).

### 3.3. Factors Influencing Referral Decisions for Cardiopulmonary Rehabilitation (Patient-Related)

**Figure 1:**
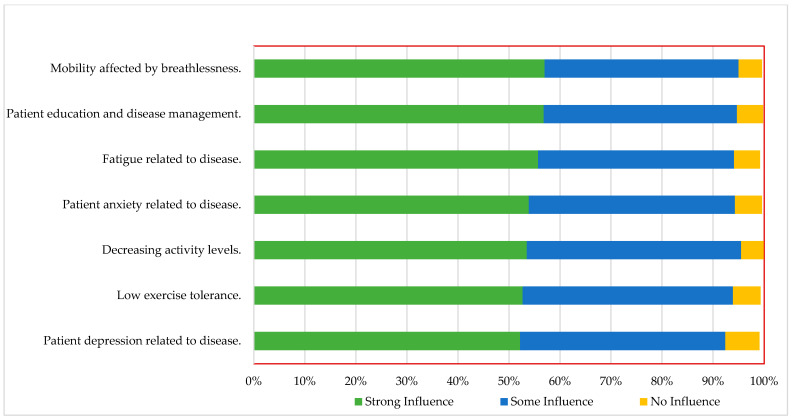
Patient-related factors that influence referral decisions for cardiopulmonary rehabilitation, using strong, some, or no influence as grading tools (*n* = 1056).

Nurses perceived mobility affected by breathlessness (57%), followed by patient education and disease management (56.80%) and fatigue related to disease (55.70%), as the most common influencing factors that strongly affect the decision to refer patients with HF for CR (Figure 1).

### 3.4. Cardiopulmonary Rehabilitation Referral Barriers

**Figure 2:**
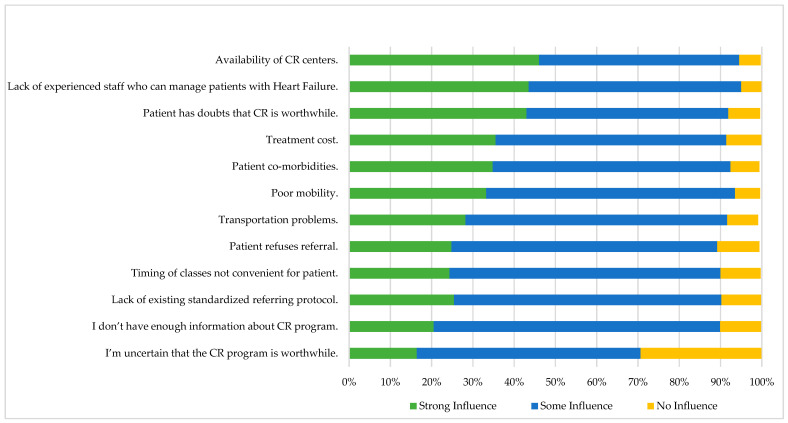
Barriers affecting decisions to refer patients with heart failure for cardiopulmonary rehabilitation, using influence graded as no, some, or strong influence (*n* = 1056).

From the nurses’ perspective, the main barriers that strongly affect the referral process of patients with HF for CR included the availability of CR centers (46%), followed by the lack of experienced staff who can manage patients with HF (43.50%) and the patients’ doubts about whether or not CR is worthwhile (43%) (Figure 2).

## 4. Discussion

To the best of our knowledge, this is the first study in Saudi Arabia that assesses attitudes toward and perceptions of the delivery of CR to HF patients and seeks to identify factors and barriers that might affect referral decisions from the nurses’ perspective. Our findings show that nurses perceived CR as an effective management strategy for patients with HF. While a home CR program was seen as the preferred mode of delivery, the lack of CR centers and well-trained staff posed significant barriers to CR referrals. Nurses perceived symptom management, followed by stress and weight management, as the most essential components of a CR program following the exercise component.

CR has been proven to be an effective and safe non-pharmacological management strategy for mitigating HF disease-related symptoms, such as fatigue and shortness of breath, in HF patients [21,22]. It also enhances patients’ exercise capacity, cardiopulmonary fitness, and quality of life by reducing HF-related hospitalization [23,24]. In our study, nurses perceived CR as an effective management strategy for improving patients’ physical activity and disease-related symptoms and reducing the hospital readmission of patients with HF.

The recent COVID-19 pandemic, with its subsequent lockdowns, curfews, and social restrictions, temporarily disrupted face-to-face CR programs. This situation may have affected the current issues with referrals or effective implementation of the programs. In the context of Saudi Arabia, a home-based program has become the most suitable option for CR, given the lack of centers available, accessibility, current infrastructure, and workforce of CR programs or centers. Home-based CR is shown to be as efficient and beneficial as the standard CR program in improving symptoms, especially the exercise capacity and dyspnea [25,26]. Simultaneously, healthcare facilities may use the current resources, even if they are limited, to establish standardized home-based CR programs.

Our study reported that the most common barrier affecting the referral of HF patients for CR from the perspectives of nurses was the lack of CR centers. There is only one CR center in the Kingdom of Saudi Arabia specifically for patients with cardiovascular diseases, including HF [16,27]. This scenario shows and highlights the urgent need or requirement to create and develop new CR programs that meet global standards and can help existing HF patients across the country. The significant gap in the application or practice of such programs requires crucial consideration and action. Given the current scenario, CR can be delivered within the current existing hospital infrastructure [28]. Ward et al. reported that an outpatient rehabilitation program offered at a small hospital was as effective as that provided by a large hospital [29]. Other CR program models which can be delivered include community-based or in-patient-based models [29,30].

Our study reported that the lack of staff or workforce with sufficient training or experience with HF patients was the second most common barrier to referral from the perspectives of nurses. Saudi Arabia is affected by healthcare staff shortages, which may limit the care of HF patients and the establishment of further rehabilitation programs [31]. Previous evidence shows that there is a lack of healthcare providers, and the number of specialized nurses is even lower in Saudi Arabia [31,32]. Additionally, only a small number of locations, programs, and disciplines are capable of managing patients with HF. However, studies have shown that using a multidisciplinary or integrative approach to patients’ management is superior [31,33]. Reduced or lack of awareness concerning HF patient management, including the lack of knowledge about the effectiveness of the interdisciplinary strategies, may explain the shortage of specialized healthcare professionals tackling HF and CR [34]. To develop and bridge the gap, government authorities in Saudi Arabia should implement incentives to the current healthcare workforce to develop skills or undertake training regarding HF, cardiovascular health, and CR. Another option is to offer high-quality education by creating programs that support international studies for the sake of encouraging specialization in cardiorespiratory management and medicines.

In our study, nurses perceived symptoms, stress, and weight management as essential components that need to be implemented within the CR programs, in addition to physical training. This is consistent with the current American College of Cardiology (ACC), American Heart Association (AHA), Heart Failure Society of America (HFSA), and British Association for Cardiovascular Prevention and Rehabilitation (BACPR) clinical guidelines regarding the main components of CR [35,36]. Patients living with HF have limited knowledge about how to manage their symptoms and the stress caused by worsening symptoms, which could be a leading factor in hospital readmission and decreased quality of life [37,38,39,40]. Therefore, promoting patient education is essential, as it may help in the management of HF-related symptoms and to improve overall health and well-being.

### Limitations

Some limitations of this study should be highlighted. The study was based on a convenience sampling strategy, which might have caused a potential selection bias. Secondly, the study did not include other healthcare professionals who may be involved in the care of patients with HF. Moreover, it would have benefited from the use of qualitative interviews to gather greater in-depth information regarding the barriers to CR programs.

## 5. Conclusions

Nurses showed their agreement on the effectiveness of CR in improving clinical outcomes. A home-based program, with symptom management being an essential component, was the preferred mode of CR delivery. The lack of CR centers was a significant barrier to the referral of patients with HF.

## Figures and Tables

**Table 1:** Demographic data and characteristics of study respondents (*n* = 1056).

Demographic Variables	Frequency (%)
Gender	
Male	395 (37.40%)
Female	661 (66.60%)
Geographical location	
Western region	320 (30.30%)
Central region	280 (26.51%)
Eastern region	195 (18.50%)
Southern region	162 (15.34%)
Northern region	99 (9.35%)
Year of experience with heart failure patients	
<1 year	27 (2.60%)
1–2 years	277 (26.20%)
3–4 years	256 (24.20%)
5–6 years	228 (21.60%)
7–8 years	133 (12.60%)
9–10 years	42 (4.00%)
>10 years	93 (8.80%)
Responsibilities for the care of patients with heart failure	
Urgent Assessments	648 (61.40%)
Primary care	629 (59.60%)
In-patient treatment	525 (49.70%)
Medication Check	521 (49.10%)
Oxygen Therapy	502 (47.50%)
Ongoing Management	477 (45.20%)
Admission Prevention	337 (31.90%)
Non-urgent Care	270 (25.60%)
Prescribing	231 (21.90%)
Diagnosis	196 (18.60%)
Outpatient clinics	134 (12.70%)
Others	23 (2.20%)

Data are presented as frequencies and percentages.

**Table 2:** Perception on referring patients with heart failure for cardiopulmonary rehabilitation (*n* = 1056).

Item	Frequency (%)
Perception on referring patients with HF for CR	
I believe CR will improve patients’ physical fitness	
Strongly agree	414 (39.20%)
Agree	438 (41.50%)
Neutral	141 (13.40%)
Disagree	33 (3.10%)
Strongly disagree	30 (2.80%)
I believe CR will reduce patients’ breathlessness	
Strongly agree	392 (37.10%)
Agree	427 (40.40%)
Neutral	166 (15.70%)
Disagree	53 (5.00%)
Strongly disagree	18 (1.70%)
I believe CR will improve patients’ palpitation and fatigue	
Strongly agree	381 (36.10%)
Agree	400 (37.90%)
Neutral	198 (18.80%)
Disagree	60 (5.70%)
Strongly disagree	17 (1.60%)
I believe CR will improve patients’ ability to perform daily activities	
Strongly agree	396 (37.50%)
Agree	427 (40.40%)
Neutral	158 (15%)
Disagree	56 (5.30%)
Strongly disagree	19 (1.80%)
I believe CR will improve patients’ ability to perform daily activities	
Strongly agree	326 (30.90%)
Agree	368 (34.80%)
Neutral	202 (19.10%)
Disagree	95 (9%)
Strongly disagree	65 (6.20%)

Data are presented as frequencies and percentages. Abbreviations: HF, heart failure; CR, cardiopulmonary rehabilitation.

**Table 3:** Mode of delivery and component of cardiopulmonary rehabilitation (*n* = 1056).

Item	Frequency (%)
The preferred way of delivering CR program to HF patients	
At home.	607 (57.50%)
In hospital.	571 (54.10%)
Tailored program with HCP support through the phone.	265 (25.10%)
Online program with healthcare provider support.	525 (49.10%)
Essential Component of CR program	
Symptom management.	704 (66.70%)
Stress management.	698 (66.10%)
Weight management.	691 (65.40%)
Smoking cessation.	644 (61%)
Information about heart failure disease.	639 (60.50%)
Information about medications.	607 (57.50%)
Others.	6 (0.60%)

Data are presented as frequencies and percentages. Abbreviations: HF, heart failure; CR, cardiopulmonary rehabilitation.

## Data Availability

The data presented and analyzed in this study are available upon request from the corresponding author.

## References

[1] Heidenreich P.A., Bozkurt B., Aguilar D., Allen L.A., Byun J.J., Colvin M.M., Deswal A., Drazner M.H., Dunlay S.M., Evers L.R. (2022). 2022 AHA/ACC/HFSA Guideline for the Management of Heart Failure: Executive Summary: A Report of the American College of Cardiology/American Heart Association Joint Committee on Clinical Practice Guidelines. Circulation.

[2] Bozkurt B., Fonarow G.C., Goldberg L.R., Guglin M., Josephson R.A., Forman D.E., Lin G., Lindenfeld J., O’Connor C., Panjrath G. (2021). Cardiac Rehabilitation for Patients With Heart Failure: JACC Expert Panel. J. Am. Coll. Cardiol..

[3] Leonardi S., Montalto C., Carrara G., Casella G., Grosseto D., Galazzi M. (2022). Clinical governance of patients with acute coronary syndromes. Eur. Heart J. Acute Cardiovasc. Care.

[4] Piña I.L., Apstein C.S., Balady G.J., Belardinelli R., Chaitman B.R., Duscha B.D. (2003). Exercise and heart failure: A statement from the American Heart Association Committee on exercise, rehabilitation, and prevention. Circulation.

[5] Forman D.E., Sanderson B.K., Josephson R.A., Raikhelkar J., Bittner V. (2015). American College of Cardiology’s Prevention of Cardiovascular Disease Section. Heart Failure as a Newly Approved Diagnosis for Cardiac Rehabilitation: Challenges and Opportunities. J. Am. Coll. Cardiol..

[6] Cooper L.B., Hernandez A.F. (2015). Assessing the Quality and Comparative Effectiveness of Team-Based Care for Heart Failure: Who, What, Where, When, and How. Heart Fail. Clin..

[7] O’Connor C.M., Whellan D.J., Lee K.L., Keteyian S.J., Cooper L.S., Ellis S.J. (2009). Efficacy and safety of exercise training in patients with chronic heart failure: HF-ACTION randomized controlled trial. JAMA.

[8] Beckers P.J., Denollet J., Possemiers N.M., Wuyts F.L., Vrints C.J., Conraads V.M. (2008). Combined endurance-resistance training vs. endurance training in patients with chronic heart failure: A prospective randomized study. Eur. Heart J..

[9] Mandic S., Tymchak W., Kim D., Daub B., Quinney H.A., Taylor D., Al-Kurtass S., Haykowsky M.J. (2009). Effects of aerobic or aerobic and resistance training on cardiorespiratory and skeletal muscle function in heart failure: A randomized controlled pilot trial. Clin. Rehabil..

[10] Tyni-Lenné R., Gordon A., Europe E., Jansson E., Sylven C. (1998). Exercise-based rehabilitation improves skeletal muscle capacity, exercise tolerance, and quality of life in both women and men with chronic heart failure. J. Card. Fail..

[11] Davies E.J., Moxham T., Rees K., Singh S., Coats A.J., Ebrahim S., Lough F., Taylor R.S. (2010). Exercise training for systolic heart failure: Cochrane systematic review and meta-analysis. Eur. J. Heart Fail..

[12] Haykowsky M.J., Timmons M.P., Kruger C., McNeely M., Taylor D.A., Clark A. (2013). Meta-analysis of aerobic interval training on exercise capacity and systolic function in patients with heart failure and reduced ejection fractions. Am. J. Cardiol..

[13] Santos F.V., Chiappa G.R., Ramalho S.H.R., de Lima A.C.G.B., de Souza F.S.J., Cahalin L.P., Durigan J.L.Q., de Castro I., Cipriano G. (2018). Resistance exercise enhances oxygen uptake without worsening cardiac function in patients with systolic heart failure: A systematic review and meta-analysis. Heart Fail. Rev..

[14] Taylor R.S., Walker S., Ciani O., Warren F., Smart N.A., Piepoli M., Davos C.H. (2019). Exercise-based cardiac rehabilitation for chronic heart failure: The EXTRAMATCH II individual participant data meta-analysis. Health Technol. Assess..

[15] Kitzman D.W., Brubaker P.H., Herrington D.M., Morgan T.M., Stewart K.P., Hundley W.G. (2013). Effect of endurance exercise training on endothelial function and arterial stiffness in older patients with heart failure and preserved ejection fraction: A randomized, controlled, single-blind trial. J. Am. Coll. Cardiol..

[16] Balady G.J., Ades P.A., Bittner V.A., Franklin B.A., Gordon N.F., Thomas R.J. (2011). Referral, enrollment, and delivery of cardiac rehabilitation/secondary prevention programs at clinical centers and beyond: A presidential advisory from the American Heart Association. Circulation.

[17] Aldhahir A.M., Alhotye M., Alqahtani J.S., AlDraiwiesh I.A., Alghamdi S.M., Alsulayyim A.S., Alqarni A.A., Khormi S.K., Alzahrani E.M., Al Rajeh A.M. (2022). Physiotherapists’ Attitudes, and Barriers of Delivering Cardiopulmonary Rehabilitation for Patients with Heart Failure in Saudi Arabia: A Cross-Sectional Study. J. Multidiscip. Health.

[18] Rashed M., Theruvan N., Gad A., Shaheen H., Mosbah S. (2020). Cardiac Rehabilitation: Future of Heart Health in Saudi Arabia, a Perceptual View. World J. Cardiovasc. Dis..

[19] Aldhahir A.M., Alqahtani J.S., Alghamdi S.M., Alqarni A.A., Khormi S.K., Alwafi H., Samannodi M., Siraj R.A., Alhotye M., Naser A.Y. (2022). Physicians’ Attitudes, Beliefs and Barriers to a Pulmonary Rehabilitation for COPD Patients in Saudi Arabia: A Cross-Sectional Study. Healthcare.

[20] Spruit M.A., Singh S.J., Garvey C., ZuWallack R., Nici L., Rochester C., Hill K., Holland A.E., Lareau S.C., Man W.D.-C. (2013). An official American Thoracic Society/European Respiratory Society statement: Key concepts and advances in pulmonary rehabilitation. Am. J. Respir. Crit. Care Med..

[21] Hill K., Vogiatzis I., Burtin C. (2013). The importance of components of pulmonary rehabilitation, other than exercise training, in COPD. Eur. Respir. Rev..

[22] Holland A.E., Cox N.S., Houchen-Wolloff L., Rochester C.L., Garvey C., ZuWallack R., Nici L., Limberg T., Lareau S.C., Yawn B.P. (2021). Defining Modern Pulmonary Rehabilitation. An Official American Thoracic Society Workshop Report. Ann. Am. Thorac. Soc..

[23] Yancy C.W., Jessup M., Bozkurt B., Butler J., Casey D.E., Drazner M.H. (2013). 2013 ACCF/AHA guideline for the management of heart failure: A report of the American College of Cardiology Foundation/American Heart Association Task Force on Practice Guidelines. J. Am. Coll. Cardiol..

[24] McDonagh T.A., Metra M., Adamo M., Gardner R.S., Baumbach A., Böhm M., Burri H., Butler J., Čelutkienė J., Chioncel O. (2021). 2021 ESC Guidelines for the diagnosis and treatment of acute and chronic heart failure: Developed by the Task Force for the diagnosis and treatment of acute and chronic heart failure of the European Society of Cardiology (ESC) With the special contribution of the Heart Failure Association (HFA) of the ESC. Eur. Heart J..

[25] Long L., Mordi I.R., Bridges C., Sagar V.A., Davies E.J., Coats A.J., Dalal H., Rees K., Singh S.J., Taylor R.S. (2019). Exercise-based cardiac rehabilitation for adults with heart failure. Cochrane Database Syst. Rev..

[26] Taylor R.S., Long L., Mordi I.R., Madsen M.T., Davies E.J., Dalal H. (2019). Exercise-based rehabilitation for heart failure: Cochrane systematic review, meta-analysis, and trial sequential analysis. JACC Heart Fail..

[27] Fanget M., Bayle M., Labeix P., Roche F., Hupin D. (2022). Effects of Cardiac Telerehabilitation During COVID-19 on Cardiorespiratory Capacities in Patients With Coronary Artery Disease. Front. Physiol..

[28] Cavalheiro A.H., Silva Cardoso J., Rocha A., Moreira E., Azevedo L.F. (2021). Effectiveness of Tele-rehabilitation Programs in Heart Failure: A Systematic Review and Meta-analysis. Health Serv. Insights.

[29] Aldhahir A.M., Alghamdi S.M., Alqahtani J.S., Alqahtani K.A., Al Rajah A.M., Alkhathlan B.S., Singh S.J., Mandal S., Hurst J.R. (2021). Pulmonary rehabilitation for COPD: A narrative review and call for further implementation in Saudi Arabia. Ann. Thorac. Med..

[30] Jenkins S., Hill K., Cecins N.M. (2010). State of the art: How to set up a pulmonary rehabilitation program. Respirology.

[31] Ward J.A., Akers G., Ward D.G., Pinnuck M., Williams S., Trott J., Halpin D.M.G. (2002). Feasibility and effectiveness of a pulmonary rehabilitation programme in a community hospital setting. Br. J. Gen. Pract..

[32] Maltais F., Bourbeau J., Shapiro S., Lacasse Y., Perrault H., Baltzan M. (2008). Effects of home-based pulmonary rehabilitation in patients with chronic obstructive pulmonary disease: A randomized trial. Ann. Intern. Med..

[33] Alsubaiei M.E., Cafarella P.A., Frith P.A., McEvoy R.D., Effing T.W. (2018). Factors influencing management of chronic respiratory diseases in general and chronic obstructive pulmonary disease in particular in Saudi Arabia: An overview. Ann. Thorac. Med..

[34] Aboshaiqah A. (2016). Strategies to address the nursing shortage in Saudi Arabia. Int. Nurs. Rev..

[35] Kuzma A.M., Meli Y., Meldrum C., Jellen P., Butler-Lebair M., Koczen-Doyle D., Rising P., Stavrolakes K., Brogan F. (2008). Multidisciplinary care of the patient with chronic obstructive pulmonary disease. Proc. Am. Thorac. Soc..

[36] Alsubaiei M.E., Frith P.A., Cafarella P.A., Quinn S., Al Moamary M.S., McEvoy R.D., Effing T.W. (2017). COPD care in Saudi Arabia: Physicians’ awareness and knowledge of guidelines and barriers to implementation. Int. J. Tuberc. Lung Dis..

[37] Cowie A., Buckley J., Doherty P., Furze G., Hayward J., Hinton S. (2019). British Association for Cardiovascular Prevention and Rehabilitation (BACPR). Standards and core components for cardiovascular disease prevention and rehabilitation. Heart.

[38] Yancy C.W., Jessup M., Bozkurt B., Butler J., Casey D.E., Colvin M.M., Drazner M.H., Filippatos G.S., Fonarow G.C., Givertz M.M. (2017). 2017 ACC/AHA/HFSA focused update of the 2013 ACCF/AHA guideline for the management of heart failure: A report of the American College of Cardiology/American Heart Association Task Force on Clinical Practice Guidelines and the Heart Failure Society of America. J. Am. Coll. Cardiol..

[39] Strömberg A. (2005). The crucial role of patient education in heart failure. Eur. J. Heart Fail..

[40] Michalsen A., Konig G., Thimme W. (1998). Preventable causative factors leading to hospital admission with decompensated heart failure. Heart.

